# Vietnamese expert consensus on the treatment of hepatocellular carcinoma with transarterial chemoembolization

**DOI:** 10.3389/fradi.2026.1763042

**Published:** 2026-04-21

**Authors:** Vu Dang Luu, Do Dang Tan, Pham Minh Thong, Le Van Khang, Le Thanh Dung, Le Trong Binh, Nguyen Ngoc Trang, Nguyen Ngoc Cuong, Nguyen Dinh Luan, Ngo Le Lam, Trinh Ha Chau, Than Van Sy, Nguyen Duy Anh, Le Duc Tho, Tran Duc Huy, Dang Ngoc Hieu, Pham Quang Son, Le Doan Tri, Nguyen Dinh Huong, Pham Cam Phuong, Nguyen Tien Thinh, Vo Duy Thong, Ho Tan Phat, Phan Thi Hong Duc, Pham Gia Anh, Vo Hoi Trung Truc, Lam Quoc Trung, Le Ba Thao, Le Tuan Anh, Tran Cong Duy Long

**Affiliations:** 1 Department of Diagnostic Imaging, Hanoi Medical University, Hanoi, Vietnam; 2 Institute for Radiology, Bach Mai Hospital, Hanoi, Vietnam; 3 Center for Diagnostic Imaging, Viet Duc University Hospital, Hanoi, Vietnam; 4 Department of Diagnostic Imaging, Hue University of Medicine and Pharmacy, Hue, Vietnam; 5 Department of Diagnostic Imaging, Hanoi Medical University Hospital, Hanoi, Vietnam; 6 Department of General Surgery, Nhan Dan Gia Dinh Hospital, Ho Chi Minh City, Vietnam; 7 Center for Diagnostic Imaging, National Cancer Hospital (K Hospital), Hanoi, Vietnam; 8 Department of Diagnostic Imaging, Hanoi Oncology Hospital, Hanoi, Vietnam; 9 Center for Nuclear Medicine and Oncology, Bach Mai Hospital, Hanoi, Vietnam; 10 Central Military Hospital 108, Hanoi, Vietnam; 11 Department of Gastroenterology, University Medical Center, University of Medicine and Pharmacy at Ho Chi Minh City, Ho Chi Minh City, Vietnam; 12 Department of Gastroenterology, Cho Ray Hospital, Ho Chi Minh City, Vietnam; 13 Department of Gastroenterology, Breast, and Urology, Ho Chi Minh City Oncology Hospital, Ho Chi Minh City, Vietnam; 14 Department of Oncology and Radiotherapy, Viet Duc University Hospital, Hanoi, Vietnam; 15 Department of Liver Tumors, Cho Ray Hospital, Ho Chi Minh City, Vietnam; 16 Department of Chemotherapy, University Medical Center, University of Medicine and Pharmacy at Ho Chi Minh City, Ho Chi Minh City, Vietnam; 17 Center for Oncology, Cho Ray Hospital, Ho Chi Minh City, Vietnam; 18 Department of Hepatobiliary and Pancreatic Surgery, University Medical Center, University of Medicine and Pharmacy at Ho Chi Minh City, Ho Chi Minh City, Vietnam

**Keywords:** consensus guidelines, hepatocellular carcinoma, interventional radiology, transarterial chemoembolization, Vietnam

## Abstract

Hepatocellular carcinoma (HCC) remains a major public health challenge in Vietnam and, according to GLOBOCAN 2022, accounted for 24,502 new cases in 2022, making it the second most common cancer and the leading cause of cancer-related mortality. Transarterial chemoembolization (TACE), introduced in 1977, is a pivotal treatment for unresectable HCC, delivering chemotherapeutic agents and embolic materials to induce tumor necrosis through cytotoxicity and ischemia. TACE is versatile and, in selected early-stage HCC (BCLC 0-A) treated with a highly selective approach, may achieve complete response; in intermediate-stage disease (BCLC B), it can prolong survival and enable downstaging, and in advanced-stage disease (BCLC C), it may complement systemic therapy. Despite its global use, TACE practices vary, and Vietnam lacks standardized guidelines tailored to its unique clinical landscape. The Vietnamese Society of Radiology and Nuclear Medicine and The Vietnamese Society of Interventional Radiology conducted three workshops in Da Nang, An Giang, and Can Tho between August and December 2024. These workshops engaged interventional radiologists from major hospitals and regional centers to develop consensus-based TACE recommendations. Through literature reviews, discussions, and physician voting, the recommendations cover pre-TACE diagnostic imaging, patient selection criteria, TACE techniques, choice of embolic materials and chemotherapeutic agents, intra- and post-procedure monitoring, and integration with combination therapies. This initiative aligns international standards with local needs, aiming to enhance the efficacy and consistency of TACE for HCC management in Vietnam.

## 1 Introduction

Hepatocellular carcinoma (HCC) is the sixth most common cancer worldwide and the third leading cause of cancer-related mortality, as reported by GLOBOCAN 2022 (International Agency for Research on Cancer). In Vietnam, HCC accounted for 24,502 new cases in 2022, ranking it as the second most prevalent cancer across all genders, the most common in males, and the leading cause of cancer mortality (1). Treatment options for HCC include curative approaches such as surgical resection and local ablation, as well as locoregional therapies like transarterial chemoembolization (TACE), selective internal radiation therapy (SIRT), external beam radiation, and hepatic arterial infusion chemotherapy (HAIC), alongside systemic therapies such as targeted agents and immunotherapy. Introduced by Yamada et al. in 1977, TACE has emerged as a primary treatment for unresectable HCC by delivering chemotherapeutic agents and embolic materials through the hepatic artery, inducing tumor necrosis via cytotoxicity and ischemia (2). TACE is versatile and, in selected early-stage HCC (BCLC 0-A) treated with a highly selective approach, may achieve complete response; in intermediate-stage disease (BCLC B), it can prolong survival and enable downstaging, and in advanced-stage disease (BCLC C), it may complement systemic therapy.

Although TACE is widely used worldwide, its indications, technical approaches, embolic materials, treatment endpoints, and integration with other therapies remain heterogeneous across countries and institutions. Differences between Western and Asian recommendations, as well as variations in local expertise and resource availability, have contributed to substantial diversity in real-world practice. In Vietnam, adaptation of international evidence to daily clinical practice is particularly challenging because HCC care is delivered across a heterogeneous healthcare system, with marked differences in infrastructure, imaging support, embolic materials, navigation software, and interventional radiology experience between tertiary referral centers and provincial hospitals. In addition, TACE practice in Vietnam remains variable among institutions, reflecting differences in operator experience, technical resources, and multidisciplinary coordination. These factors, together with the high national burden of HCC, underscore the need for practical recommendations adapted to the Vietnamese setting.

The objective of this manuscript is to develop expert consensus recommendations for the use of TACE in HCC in Vietnam. These recommendations were generated through literature review, multi-center expert discussion, and physician voting, and are intended as a practical consensus document rather than an official guideline. Their aim is to support more standardized TACE practice and to provide a practical reference for interventional radiologists and multidisciplinary teams in Vietnam.

## 2 Methods

This manuscript was developed as an expert consensus document on the use of transarterial chemoembolization (TACE) for hepatocellular carcinoma (HCC) in Vietnam. Before each meeting, the secretariat reviewed and synthesized relevant literature to prepare draft content and statements on key topics requiring expert input. The literature review focused on major domains of TACE practice, including pre-treatment imaging, patient selection, technical aspects of conventional TACE (cTACE) and drug-eluting bead TACE (DEB-TACE), embolic materials, peri-procedural management, treatment response assessment, TACE refractoriness, and combination strategies. Search terms included combinations of “hepatocellular carcinoma,” “HCC,” “transarterial chemoembolization,” “TACE,” “cTACE,” “DEB-TACE,” “dynamic CT,” “MRI,” “cone-beam CT,” “tumor feeder detection,” “patient selection,” “embolization technique,” “response assessment,” “mRECIST,” “TACE refractoriness,” and “combination therapy.” Priority was given to international and regional guidelines, expert consensus statements, randomized controlled trials, meta-analyses, prospective studies, and selected multicenter retrospective studies with direct relevance to TACE practice. Case reports, very small case series, duplicate reports, and studies with limited relevance to clinical decision-making were not prioritized. Because this manuscript was intended to support expert discussion and voting, the literature review followed a structured narrative approach rather than a formal systematic review.

The consensus process involved three workshops, with a total of 40 physicians participating across all sessions. The expert panel included interventional radiologists, gastroenterologists/hepatologists, hepatobiliary-pancreatic surgeons, and medical oncologists. For each topic, draft statements were presented to the panel for discussion, revision, and consensus voting. Statements were refined according to expert feedback and finalized when they achieved agreement from more than 75% of participating experts. Statements that did not reach this threshold were revised and re-discussed until consensus was achieved.

All authors reviewed and approved the final consensus statements. The manuscript was developed based on expert discussion and literature synthesis.

## 3 Recommendations on HCC treatment with TACE

### 3.1 Diagnostic imaging modalities used before TACE

Computed tomography (CT) and magnetic resonance imaging (MRI) are critical for diagnosing HCC by evaluating tumor enhancement kinetics. These modalities are recommended as first-line tools for HCC diagnosis and staging, assessing tumor number, size, and location. Dynamic CT or MRI requires at least three phases—pre-contrast, arterial, and portal venous—with precise contrast injection rates and timing to optimize HCC detection. The late arterial phase is preferred for diagnosing HCC. For hypovascular tumors, late arterial and portal venous phases provide greater contrast between the tumor and surrounding liver parenchyma compared to the early arterial phase. For hypervascular tumors, the late arterial phase enhances visibility over the early arterial phase. Typical HCC exhibits strong arterial-phase enhancement and venous- phase washout (3–5). Early-stage HCC, supplied by both portal veins and arteries, shows reduced portal vein branches (approximately 25% of normal liver tissue) and immature arterial supply, resulting in lower vascularity than healthy parenchyma. Consequently, early-stage HCC often lacks hypervascularity on angiography or contrast-enhanced CT. On abdominal ultrasound, small early-stage HCC typically appears hyperechoic due to fatty degeneration, most frequent in tumors 10–15 mm in diameter (approximately 40%), serving as a morphological hallmark. MRI with in- phase and out-of-phase sequences aids in detecting early-stage HCC nodules (3, 6). Early-stage HCC generally exhibits low vascularity. MRI with hepatocyte-specific contrast agents assesses liver cell function, identifying HCC as non-enhancing lesions in the hepatobiliary phase while distinguishing it from benign hypervascular lesions lacking washout on standard triphasic MRI (3, 7, 8).

Contrast-enhanced ultrasound (CEUS) evaluates enhancement patterns in two phases: the vascular phase (up to 3 min post-injection) and the post-vascular (Kupffer) phase (after 10 min). In hypervascular HCC, the tumor enhances strongly in the arterial phase and washes out in the portal venous phase, with faster washout indicating lower differentiation. Kupffer-phase imaging reflects contrast uptake by Kupffer cells; hypervascular HCC, with reduced or absent Kupffer cells, appears hypo- or non-enhancing, while well-differentiated HCC with preserved Kupffer cells appears isoechoic or slightly hypoechoic relative to the surrounding liver. CEUS provides high temporal and spatial resolution, enabling simultaneous assessment of tumor hemodynamics, vascular structure, Kupffer cell presence, and morphology, outperforming other modalities in differential diagnosis (9–11). CEUS vascular and Kupffer-phase imaging correlates with histological differentiation; as differentiation decreases, vascular enhancement shifts from “smooth” to “irregular,” and the tumor-to-non-tumor echogenicity ratio in the Kupffer phase declines. Nodules with irregular vascularity warrant special attention, as they are more likely to be poorly differentiated HCC with an elevated risk of portal vein invasion. Kupffer-phase CEUS excels at detecting nodules not visible on B-mode ultrasound, identifying residual or recurrent lesions, and guiding treatment (12, 13).

The interval between pre-treatment CT/MRI and TACE should be minimized to account for potential tumor growth, serving as a baseline for response assessment. A 2014 international consensus recommended that this interval be ideally less than 1 month and not exceed 2 months (14, 15).

Pre-intervention multi-slice CT (MSCT) is essential for interventionalists, assessing morphology, anatomical variants, and major tumor-feeding arteries, including intra- and extrahepatic branches, to optimize procedural planning, reduce x-ray exposure, and enhance efficacy (16–18).

Statement 1.1:

- Dynamic computed tomography (CT) and magnetic resonance imaging (MRI) are essential for HCC diagnosis through kinetic evaluation of tumor enhancement.
- MRI with hepatocyte-specific contrast agents enhances the detection of small HCC lesions and differentiates them from benign hypervascular lesions.
- Contrast-enhanced ultrasound (CEUS) evaluates tumor kinetics, vascular architecture, and morphology, and aids in detecting post-embolization recurrence.
- Pre-intervention CT or MRI should be performed within 1 month to ensure accurate baseline assessment.
- Pre-intervention multi-slice CT (MSCT) vascular mapping delineates the morphology and anatomical variants of major arteries (e.g., celiac trunk, superior mesenteric artery, common hepatic artery), identifying extrahepatic tumor-feeding branches.

### 3.2 Patient selection for transarterial chemoembolization (TACE)

Effective patient selection for TACE maximizes therapeutic benefits, minimizes liver function deterioration, and prevents overuse. Global guidelines from Europe, the United States, and the Asia-Pacific Liver Association base selection on liver function, performance status, vascular invasion, extrahepatic metastasis, tumor number, and size (19–22). For unresectable HCC, randomized trials and meta-analyses since the 2000s have shown that TACE improves survival compared to conservative management (23–26). However, with advances in local ablation, SIRT, targeted therapies, and immunotherapy, TACE indications require refinement. Per the 2022 Barcelona Clinic Liver Cancer (BCLC) staging system and guidelines from Europe, the U.S., and Asia-Pacific regions, TACE is prioritized for patients unsuitable for surgery, with preserved liver function (Child-Pugh ≤7), good performance status, no major vascular invasion, and tumors more than three nodules, each ≥3 cm in diameter. When surgery or ablation is unfeasible, TACE serves as an alternative (19, 20) (European Association for the Study of the Liver & European Organisation for Research and Treatment of Cancer, 2012) (22). Beyond tumor characteristics, poor liver function (ALBI grade 2 or Child-Pugh B) predicts worse survival and may exacerbate post-TACE decline, limiting subsequent systemic options (27–30).

HCC management requires balancing liver function with multiple treatment modalities, underscoring the role of multidisciplinary teams (MDTs). Guidelines from AASLD, ESMO, and NCCN emphasize MDTs in TACE decision-making (20, 27). Beyond liver function and tumor features (>3 nodules, each ≥3 cm), indications should consider tumor margin definition, multilobar distribution, the “up-to-7” criterion, and HCC subgroups predictive of TACE non-response or failure. Japanese experts introduced concepts like TACE unsuitability, refractoriness, and failure in their 2021 consensus (3).

For patients with major vascular invasion, Asian guidelines support TACE, while Western guidelines contraindicate it. A 2023 trial by Peng et al. demonstrated that TACE combined with lenvatinib outperformed lenvatinib alone in advanced HCC (31). In carefully selected patients, particularly those with preserved liver function and technically feasible liver-dominant disease, TACE may still be considered within a multimodality strategy. This is especially relevant in rare but challenging situations such as hepatic vein, inferior vena cava, or right atrial tumor thrombus, where individualized treatment selection is essential, and systemic therapy sequencing may continue to evolve, including in the setting of immune checkpoint inhibitor rechallenge (32, 33).

Statement 1.2:

- Patient selection for TACE is determined by performance status, liver function, tumor number, size, vascular invasion, and extrahepatic metastasis.
- For unresectable HCC with no major vascular invasion, preserved liver function, >3 nodules, and each ≥3 cm, TACE is the preferred treatment.
- For HCC indicated for surgery or ablation but deemed unfeasible, TACE is an alternative.
- For HCC with major vascular invasion and preserved liver function, TACE may be combined with systemic therapies, guided by an MDT.
- In cases at risk of TACE failure or refractoriness, systemic therapy should be considered alone or with TACE, as determined by an MDT

### 3.3 Approach routes, support tools, and TACE techniques

The femoral artery is the conventional route for hepatic artery chemoembolization, widely familiar to interventional radiologists (34). Recent advances have popularized the radial artery approach, initially used in coronary interventions, which may reduce local vascular complications and hospital stay duration, though it demands greater technical skill and has a lower success rate than the femoral approach (35–37).

Hepatic artery anatomy varies, originating from the celiac trunk, superior mesenteric artery, or directly from the aorta (38). Thorough visualization of hepatic artery branches ensures accurate identification of tumor-feeding vessels, avoiding errors due to anatomical variants (39). Celiac artery stenosis—from atherosclerosis, median arcuate ligament syndrome, or prior interventions—is common and may require direct celiac access or indirect approaches via collateral circulation between the celiac trunk and superior mesenteric artery, depending on etiologies (40).

Arteries sharing origins with hepatic arteries (e.g., right gastric, falciform, cystic) supply other organs, and their identification prevents extrahepatic complications like gastritis, skin necrosis, or cholecystitis post—TACE. Extrahepatic arteries (e.g., inferior phrenic, intercostal, internal mammary, renal, or right adrenal) may also feed HCC, necessitating precise recognition to ensure effective treatment and minimize recurrence (38, 41).

Cone-beam CT, hybrid Angio-CT systems, and navigation software improve accuracy in HCC detection, diagnosis, and identification of tumor-feeding arteries for treatment planning (3, 42). Studies demonstrate that Cone-beam CT and navigation software reduce procedure time, radiation exposure, and enhance diagnostic precision of tumor-feeding vessels compared to standard digital subtraction angiography (DSA) (43, 44).

Selective embolization minimizes chemotherapeutic and embolic material doses, reducing liver damage while enhancing tumor treatment efficacy (45). Superselective embolization targets subsegmental hepatic branches, and microcatheters ≤2 Fr enable curative outcomes in conventional TACE (cTACE) and optimize drug-eluting bead TACE (DEB-TACE) efficacy (45, 46). When feasible, selective embolization is preferred over non-selective approaches.

Statement 1.3:

- Hepatic artery chemoembolization may be performed via the femoral artery or alternative routes.
- All tumor-feeding arteries, including their origins, anatomical variants, extrahepatic supply, and collateral circulation, must be evaluated.
- Support tools such as Cone-beam CT, Angio-CT, and navigation software may enhance TACE precision.
- Superselective embolization with microcatheters ≤2.0 Fr is preferred to maximize treatment efficacy.

### 3.4 Embolic materials and chemotherapeutic agents

Recent studies from Korea and Japan demonstrate that superselective cTACE outperforms bland embolization (47, 48). A Korean prospective multicenter study found DEB- TACE most effective for tumors 2–5 cm, while cTACE excelled for tumors <2 cm (49). Retrospective multicenter studies in Korea and a Japanese randomized trial confirmed cTACE's superiority over DEB-TACE for tumors <3 cm (47, 48).

Common chemotherapeutic agents in cTACE include doxorubicin, famorubicin, and cisplatin, with maximum per-session doses of 75 mg for doxorubicin and famorubicin, and 2 mg/kg (up to 200 mg) for cisplatin. Differences in tumor response between agents remain unclear. Powder formulations are preferred, mixed with contrast at 10 mg/0.5 mL. For cisplatin (available only in solution), infusion via microcatheter into the hepatic artery followed by TACE is recommended, with dose reduction or avoidance in renal impairment (50, 51).

Lipiodol's maximum dose is 15 mL, as >20 mL risks pulmonary embolism or dyspnea (52). A “water-in-oil” emulsion stabilizes chemotherapeutic agents within lipiodol for delivery to the tumor's vascular bed, with contrast preferred over saline for mixing to increase specific gravity. Gelatin sponge is recommended for occluding feeding arteries (53, 54) widely used in Korea, unlike Western practices. Smaller, spherical sponge particles reach distal arteries, potentially enhancing efficacy, but may increase risks of biliary damage, parenchymal injury, and systemic embolization, particularly with large tumors. Temporary embolic materials require careful consideration of tumor size, feeding artery size, and microcatheter position.

DEB-TACE employs drug-eluting microspheres to occlude tumor vasculature, slowly releasing chemotherapeutic agents and reducing systemic exposure. Randomized trials show no significant differences between DEB-TACE and cTACE in tumor response, time to progression, or liver toxicity (55, 56). However, DEB-TACE patients report less pain, milder post-embolization syndrome, and shorter hospital stays (56). Non-selective DEB-TACE may cause biliary injury, though superselective DEB-TACE shows minimal toxicity differences from cTACE (48). Microsphere size (100–300 µm) selection depends on tumor size and feeding vessel diameter, with better responses than larger bead (57, 58). A multicenter study from South Korea reported that drug-eluting bead transarterial chemoembolization (DEB-TACE) achieved the highest response rates in tumors measuring 2–5 cm in diameter, with reduced efficacy in lesions ≤2 cm (49). Similarly, a retrospective multicenter study from South Korea and a randomized controlled trial from Japan demonstrated that DEB-TACE exhibited a lower response rate than conventional TACE (cTACE) in tumors ≤3 cm (47, 48). This difference may be attributed to smaller tumor-feeding arteries in smaller tumors, which impede the delivery of relatively large microspheres compared to liquid agents such as Lipiodol. Notably, these studies utilized DEB microspheres ranging from 100 to 300 µm in size. Further research is warranted to evaluate the efficacy of newer microspheres ≤150 µm. Experts recommend cTACE for tumors within the “up-to-7” criterion (3–6 cm, <4 nodules) and DEB- TACE for larger tumors or poor liver function. Combining DEB-TACE and cTACE may be tailored to individual cases.

In cases of advanced hepatocellular carcinoma (HCC) with portal vein invasion or exceeding the “up- to-7” criterion, radioembolization using yttrium-90 (Y90) microspheres demonstrates superior response rates and survival outcomes compared to transarterial chemoembolization (TACE) or sorafenib monotherapy (59, 60). The radioactive microspheres travel through small arterial branches, distributing throughout the tumor and occluding its feeding vessels. Additionally, the low-energy radiation emitted by Y90 destroys cancer cells and induces fibrosis in the tumor-feeding vasculature. This results in a reduction of tumor volume or complete tumor eradication within the liver, with minimal impact on surrounding healthy tissue. A key advantage of this technique is its ability to deliver high radiation doses to the tumor while exposing adjacent healthy tissue to low doses, thereby minimizing side effects and reducing treatment-related complications.

Statement 1.4:

- Selection of cTACE, DEB-TACE, or Combination Therapy

The choice of conventional transarterial chemoembolization (cTACE), drug-eluting bead TACE (DEB- TACE), or a combination depends on tumor vascularity, size, individual patient characteristics, and facility capabilities:

– cTACE Indication: Preferred for tumors within the “up-to-7” criterion (tumor diameter 3–6 cm, fewer than 4 nodules).
– DEB-TACE Indication: Preferred for tumors outside the “up-to-7” criterion (tumor diameter 3–6 cm, fewer than 4 nodules) or in cases of impaired liver function (Child-Pugh B8 or B9).
– cTACE + DEB-TACE Combination: Applied on a case-by-case basis.

- Chemotherapeutic Agents

Commonly used agents include doxorubicin, epirubicin, famorubicin, cisplatin, 5-fluorouracil (5-FU), and absolute alcohol, with dosages determined by patient weight and body surface area. Powder formulations are preferred.

- Temporary Embolic Materials

Occlusion of tumor-feeding arteries with temporary embolic materials is recommended following cTACE.

- Lipiodol Use in cTACE

Lipiodol is utilized in cTACE with a maximum dose of 15 mL per session, mixed with chemotherapeutic agents at a ratio of 2:1–4:1, prepared according to proper technique.

- Radioembolization with Microspheres

Radioembolization using microspheres is applicable across various HCC stages, including advanced cases with portal vein or hepatic vein invasion.

- Additional Recommendations for DEB-TACE
  – Microsphere Size Selection: Choose bead size based on tumor size, degree of selectivity, and microcatheter size; beads ranging from 100 to 300 µm are effective.
  – Chemotherapeutic Agent Selection: Select agents compatible with drug-eluting beads; doxorubicin is commonly used.
  – Microcatheter Size Selection: Determine microcatheter size (typically 1.7–2.4 Fr) based on the degree of selectivity and intended bead size.
  – Selective Approach: Prioritize selective access to subsegmental hepatic artery branches during DEB-TACE.
  – Extrahepatic Feeding Arteries: DEB-TACE may be used for extrahepatic tumor-feeding arteries if superselective access is achieved.
  – Arteriovenous Shunts: DEB-TACE is feasible in cases with hepatic arteriovenous shunts after shunt control.
  – Bead Preparation: Prepare drug-eluting beads prior to the procedure following the manufacturer's guidelines.
  – Injection Technique: Mix beads with contrast medium and inject under digital subtraction angiography (DSA) guidance to prevent reflux (typically at 1 mL/min)
  – Endpoint of Injection: Cease injection when near-stasis is achieved (no re-flow after 5 heartbeats), reassess after 5 min, and consider additional injection for deformable beads.
  – Combination with cTACE: cTACE may be combined with DEB-TACE as needed.
  – Management of Intratumoral Vessel Rupture: If vessel rupture occurs during bead injection, halt the procedure and manage according to hematoma progression. Embolize the ruptured branch with temporary materials (e.g., Lipiodol, Gelfoam) or permanent materials (e.g., glue, coils), and proceed to treat another artery if bleeding is controlled.

### 3.4 Monitoring during and after intervention, response assessment, TACE refractoriness, and decision to discontinue TACE

#### 3.4.1 Before and during intervention

Routine prophylactic antibiotics are not recommended for TACE, but high-risk patients (e.g., immunocompromised, diabetic, biliary dilatation, post-biliary stenting, bilioenteric anastomosis, sphincterotomy) should receive prophylaxis (61–64). Patients with prior biliary intervention or surgery face elevated infection risks post- TACE, necessitating antibiotics and monitoring (63, 64).

Numerous studies have demonstrated that intra-arterial lidocaine administration prior to transarterial chemoembolization (TACE) reduces both the incidence and severity of pain during and after the procedure, thereby decreasing the required dose of analgesics (65–67). Furthermore, post-procedure pain may occur even in patients who experience no discomfort during TACE, supporting the routine use of lidocaine injection before the procedure (66).

Fever, pain, nausea, and vomiting are the most common adverse effects following transarterial chemoembolization (TACE). In clinical practice, prophylactic antiemetics and anti-inflammatory drugs are frequently administered to reduce the incidence and severity of these undesirable symptoms and mitigate post-embolization syndrome. Several randomized clinical trials have demonstrated that nonsteroidal anti- inflammatory drugs (NSAIDs), such as parecoxib, can decrease pain intensity, shorten pain duration, and reduce the need for opioid analgesics (68, 69). However, caution is advised when using NSAIDs due to their potential to increase the risk of renal impairment, particularly in hepatocellular carcinoma (HCC) patients with underlying cirrhosis. Steroids may also be employed for this purpose, though their use requires careful consideration of associated adverse effects (70, 71).

Statement 1.5:

- Prophylactic antibiotics before TACE are recommended for high-risk patients (e.g., immunocompromised, diabetic, biliary dilatation, post-biliary stenting, bilioenteric anastomosis, sphincterotomy).
- Selective intra-arterial lidocaine during TACE may reduce the incidence and severity of post- embolization syndrome.
- Antiemetics and anti-inflammatory drugs alleviate intra- and post-procedure symptoms and reduce post-embolization syndrome severity

#### 3.4.2 After intervention

Post-TACE, patients may experience transient symptoms from chemotherapeutic effects, embolization of the tumor and adjacent parenchyma, and systemic drug circulation. Common adverse effects include elevated liver enzymes (18.1%), fever (17.2%), bone marrow suppression (13.5%), pain (11%), and vomiting (6%), with a very low mortality rate. Adverse effects are classified by severity:

- Mild: Nausea, vomiting, pain, burning sensation in the liver, chest warmth, right shoulder pain, transient hypertension, fever, and post-embolization syndrome—typically resolving within hours or days. Elevated liver enzymes and occasional bilirubin increases from hepatocyte damage usually normalize within 1–2 weeks (72)
- Severe (<10% incidence): Necrosis, liver abscess, biliary complications (cholecystitis, bile leak, strictures), gastrointestinal ulcers, vascular complications (thrombosis, dissection, stenosis) (73). Liver failure occurs in 3%–5% of patients, with a 0%–4% mortality rate within 30 days (74). Post-TACE liver function assessment enables early detection and management of liver failure, reducing mortality risk. Peripheral biliary strictures are often insignificant, but central strictures may cause severe outcomes like widespread liver damage, failure, or recurrent cholangitis
- Post-Embolization Syndrome: Defined as occurring 1–3 days post-TACE with at least one of: fever >38.5 °C, nausea/vomiting, and abdominal pain requiring analgesics, plus elevated transaminases. It affects 40%–80% of cTACE patients, typically resolving within 3–4 days with vital sign monitoring and symptomatic treatment (72, 75). Inflammatory mediators from ischemia or necrosis and systemic chemotherapeutic effects contribute to its onset, with greater frequency and severity in cTACE than DEB-TACE due to higher drug washout.
- Treatment: Managed effectively with analgesics, antipyretics, antiemetics, oral gastrointestinal protection, and hydration, enabling discharge within 1–3 days.

Statement 1.6:

- Early monitoring and detection of local and systemic complications are essential post-intervention.
- Post-embolization syndrome should be treated based on symptom severity with antipyretics, analgesics, antiemetics, and supportive care.

#### 3.4.3 Response assessment after TACE

Post-TACE imaging intervals vary globally. Taiwan guidelines recommend CT/MRI at 8–10 weeks (76) while Korean consensus suggests 4–8 weeks (14). We recommend 4–8 weeks to assess response and plan subsequent treatment. Tumor markers (AFP, PIVKA-II) complement imaging, particularly when results are inconclusive.

The mRECIST and LI-RADS criteria reliably evaluate treatment response by assessing residual viable tumor enhancement, correlating with progression-free survival (PFS) and overall survival (OS) (77, 78). For complete responders, imaging and tumor marker follow-up every three months is advised.

TACE failure/refractoriness is defined as follows:

1. Intrahepatic lesions
  (a) Residual enhancement (≥50%) of treated nodules observed on response evaluation CT/MRI images obtained 1–3 months after ≥2 consecutive TACE sessions (despite changing chemotherapeutic agent or reanalyzing the feeding artery).
  (b) Increased number of intrahepatic lesions from the previous TACE session observed on response evaluation CT/MRI images obtained 1–3 months after ≥2 consecutive TACE sessions (despite changing chemotherapeutic agent, changing embolization material, or reanalyzing the feeding artery or device).
2. Tumor markers: No decrease in tumor marker level is observed immediately after TACE, or only a minimal and transient decrease is observed after TACE, immediately followed by an increasing trend.
3. Development of vascular invasion.
4. Development of extrahepatic spread

We recommend performing a minimum of two consecutive transarterial chemoembolization (TACE) sessions per treatment cycle to assess the response of hepatocellular carcinoma (HCC) to TACE. Repeated TACE is known to prolong patient survival; however, excessive TACE sessions may increase the risk of liver function decline, which can adversely affect overall survival (79). Additionally, multiple TACE procedures may heighten the risk of malignant transformation, such as sarcomatous change or mixed HCC-cholangiocarcinoma, complicating treatment in these cases (46). Furthermore, evidence indicates no survival benefit in patients unresponsive to TACE compared to untreated patients, suggesting that repeated TACE should not be pursued in the absence of an objective response (23).

For HCC cases refractory to TACE, where no objective response is achieved after at least two sessions, alternative treatments should be considered. This decision requires a detailed technical evaluation (e.g., whether all tumor-feeding arteries were fully controlled, and the type, dose, and concentration of chemotherapeutic agents used), as well as an assessment of liver function and the patient's overall condition, before discontinuing TACE (3). Prognostic factors, including tumor size, imaging response, tumor markers, and changes in Child-Pugh score, should be considered when determining subsequent treatment strategies.

Statement 1.7:

• Tumor response should be assessed 4–8 weeks post-TACE with multiphasic contrast-enhanced CT and/or MRI.
• mRECIST or LI-RADS criteria, combined with tumor marker changes, should guide response
• TACE failure/refractoriness is defined as follows:
1. Intrahepatic lesions
  (a) Residual enhancement (≥ 50%) of treated nodules observed on response evaluation CT/MRI images obtained 1–3 months after ≥ 2 consecutive TACE sessions (despite changing chemotherapeutic agent or reanalyzing the feeding artery).
  (b) Increased number of intrahepatic lesions from the previous TACE session observed on response evaluation CT/MRI images obtained 1–3 months after ≥ 2 consecutive TACE sessions (despite changing chemotherapeutic agent, changing embolization material, or reanalyzing the feeding artery or device).
2. Tumor markers: No decrease in tumor marker level is observed immediately after TACE, or only a minimal and transient decrease is observed after TACE, immediately followed by an increasing trend.
3. Development of vascular invasion.
4. Development of extrahepatic spread
  - TACE should be discontinued in cases of failure, refractoriness, or unsuitability, as decided by an MDT.

### 3.6 TACE combined with other treatments

#### 3.6.1 Surgery

Surgery is a curative option for HCC, but patient selection is complex, requiring evaluation of tumor stage, liver function, and portal hypertension. MDT consensus minimizes recurrence and complications. Studies show TACE combined with resection enhances curative outcomes, reduces recurrence, and extends survival. A study of 488 BCLC B HCC patients reported 5-year survival rates of 52.9% for TACE plus surgery vs. 33.8% for surgery alone (80).

#### 3.6.2 Local ablation

Ablation is curative for tumors ≤3 cm and ≤3 nodules, safe for patients ineligible for surgery (e.g., low platelets, coagulopathy, Child-Pugh B, bilobar tumors). Combining TACE with ablation is under study, particularly for tumors >3 cm. A study of 201 HCC patients with 3–10 cm tumors and ≤3 nodules showed TACE plus radiofrequency ablation (RFA) improved OS (27.57 vs. 12 months) and PFS (12 vs. 9.12 months) compared to TACE alone (81).

#### 3.6.3 Systemic therapy with molecular targeted agents (MTAs)

Combining TACE with MTAs is a growing trend, especially for advanced HCC, due to:

- *MTA Antitumor Effects:* MTAs inhibit angiogenesis and tumor growth in untreated lesions, preventing progression of residual tumors, new intrahepatic lesions, vascular invasion, and metastases.
- *Vascular Effects:* MTAs normalize tumor vasculature, reducing interstitial pressure and enhancing TACE drug delivery (82)
- *Cytokine Storm Suppression:* Post-TACE hypoxia activates HIF-1*α*, increasing VEGF and tumor progression; MTAs mitigate this

The use of molecular targeted agents (MTAs) before or immediately after transarterial chemoembolization (TACE) inhibits this increase and prevents tumor progression. These mechanisms underpin the proposed role of MTAs in combination with TACE. Concurrent MTA administration may enhance TACE efficacy, prevent progression of residual tumors, and reduce the emergence of new intrahepatic lesions, thereby extending the interval between TACE sessions (83). It may also decrease the total number of TACE procedures a patient undergoes over their lifetime, mitigating liver function decline. Additionally, combining MTAs with TACE may prolong survival by preventing vascular invasion and extrahepatic spread. Various clinical trials have been conducted based on these concepts (84–87). A notable exception is the TACTICS trial in Japan, which provides the only positive evidence supporting the efficacy of TACE combined with MTAs (87). The TACTICS trial, a phase 2 randomized study, compared sorafenib plus conventional TACE (cTACE) with TACE alone. At the study's conclusion, the median progression-free survival (PFS) was 25.2 months in the TACE plus sorafenib group vs. 13.5 months in the TACE-only group [hazard ratio [HR]: 0.59; 95% confidence interval [CI]: 0.41–0.87; *p* = 0.006], indicating a significant PFS improvement with concurrent sorafenib. The median time to extrahepatic spread or vascular invasion was 22.5 months in the TACE plus sorafenib group vs. 6.3 months in the TACE-only group (HR: 0.31; 95% CI: 0.15–0.63; *p* = 0.001), demonstrating that sorafenib significantly reduced the incidence of extrahepatic spread or vascular invasion. The median interval between TACE sessions was 21.1 weeks in the TACE plus sorafenib group vs. 16.9 weeks in the TACE-only group (*p* = 0.018), confirming that sorafenib significantly prolonged the time between TACE procedures (83).

Regarding lenvatinib-TACE outcomes, pretreatment with lenvatinib followed by TACE may improve survival in patients with bilobar tumors compared to TACE or lenvatinib alone, particularly in those unsuitable for TACE monotherapy. A recent proof-of-concept study showed that lenvatinib followed by TACE in patients not meeting the “up-to-7” criterion and ineligible for TACE yielded favorable result (88).

Combining TACE with immune checkpoint inhibitors, such as durvalumab, and the anti-angiogenic agent bevacizumab, has shown promising outcomes. The EMERALD-1 study (NCT03778957) (89) a phase 3, randomized, double-blind, multicenter trial, was the first to evaluate TACE combined with durvalumab, with or without bevacizumab (an anti-VEGF agent), in patients with unresectable hepatocellular carcinoma (HCC) still eligible for embolization. Eligible patients were randomized 1:1:1 to receive durvalumab + bevacizumab + TACE, durvalumab + TACE, or TACE alone. PFS was significantly improved with durvalumab + bevacizumab + TACE compared to TACE alone (median PFS: 15.0 vs. 8.2 months; HR: 0.77; 95% CI: 0.61–0.98; *p* = 0.032), with consistent results across most predefined subgroups. The objective response rates (ORR) were 43.6%, 41.0%, and 29.6%, and the median time to progression (mTTP) was 22.0, 11.5, and 10.0 months for durvalumab + bevacizumab + TACE, durvalumab + TACE, and TACE alone, respectively. The durvalumab +bevacizumab + TACE regimen represents the first immune checkpoint inhibitor-based combination in a global phase 3 trial to demonstrate statistically and clinically significant PFS improvement over TACE alone in patients with unresectable HCC eligible for embolization.

#### 3.6.4 Hepatic arterial infusion chemotherapy (HAIC)

HAIC treats advanced HCC but has declined with systemic therapy advances, lacking comparative studies. It is used for portal vein trunk invasion, poor liver function precluding systemic therapy, or progression post- systemic treatment.

Statement 1.8: TACE with Surgery:

- Pre-surgical TACE is recommended for intermediate-stage HCC localized to a lobe or segment, with adequate liver function and volume, as decided by MDT.
- Post-surgical TACE is advised for intermediate-stage recurrent HCC unsuitable for RFA or repeat surgery.
- Pre-surgery TACE enhances efficacy in surgical candidates.
- Post-TACE portal/hepatic vein embolization induces remnant liver hypertrophy, expanding surgical eligibility.

TACE with Ablation:

- Pre-ablation TACE is recommended for intermediate-stage HCC >3 cm, ≤3 nodules, decided by MDT.
• Post-ablation TACE is advised for recurrent intermediate-stage HCC unsuitable for ablation or surgery.
• Pre-ablation TACE reduces tumor size and localizes it, optimizing ablation efficacy and reducing recurrence.
- TACE enhances tumor visibility on ultrasound/CT, aiding RFA.

TACE with Systemic Therapy:

- TACE plus systemic therapy is recommended for intermediate-stage HCC at risk of refractoriness or advanced HCC confined to two segments with Child-Pugh A, decided by MDT.
- Combination reduces post-TACE progression and extends TACE intervals, preserving liver function.
- Systemic therapy is advised post-TACE failure or when TACE is unfeasible. HAIC Use:
- TACE may follow a good HAIC response to achieve a complete response.
- TACE with HAIC addresses extrahepatic feeding arteries, reducing HAIC efficacy.
- Non-port HAIC with TACE is suitable for high tumor burden or portal vein thrombosis.

### 3.7 Proposed level of support for consensus statements

For transparency, each consensus statement was assigned a proposed level of support based on the type and consistency of the evidence cited in the manuscript.

- Level A indicates strong support from major guidelines/consensus documents and/or randomized trials or meta-analyses;
- Level B indicates moderate support from prospective, multicenter, or otherwise consistent studies;
- Level C indicates low support mainly from retrospective, technical, or indirect evidence
- Level D indicates expert consensus or practice points based primarily on clinical experience and technical feasibility.

These proposed levels are provided for reference only and do not represent a formal guideline grading system. The proposed levels of support for the consensus statements are summarized in Tables 1–5.

**Table 1:** Proposed level of support for statements in section 3.1.

Statement	Proposed level
Multiphasic contrast-enhanced CT and MRI play a primary role in HCC diagnosis through assessment of enhancement dynamics	Level A
Hepatocyte-specific contrast-enhanced MRI can improve detection of early HCC lesions and help differentiate HCC from benign enhancing lesions	Level B
Contrast-enhanced ultrasound can assess tumor hemodynamics and morphology and may also be used to evaluate recurrence after embolization	Level B
Baseline contrast-enhanced CT and/or MRI prior to TACE should ideally be performed within 1 month before treatment	Level B
Pre-procedure multidetector CT angiographic mapping can help define arterial anatomy and variants and identify extrahepatic tumor feeders for TACE planning	Level B

**Table 2:** Proposed level of support for statements in section 3.2.

Statement	Proposed level
Patient selection for TACE should be based on performance status, liver function, tumor number, tumor size, vascular invasion, and extrahepatic metastasis	Level A
For unresectable HCC without major vascular invasion, with preserved liver function, more than three nodules, and lesions measuring at least 3 cm, TACE is generally a preferred treatment option	Level A
For HCC in which surgery or local ablation is indicated but not feasible, TACE may be considered as an alternative locoregional treatment	Level B
For HCC with major vascular invasion and preserved liver function, TACE may be considered in combination with systemic therapy in selected patients, with the decision guided by a multidisciplinary tumor board	Level B
In patients at high risk of TACE failure or refractoriness, systemic therapy should be considered either alone or in combination with TACE, as determined by a multidisciplinary tumor board	Level B

**Table 3:** Proposed level of support for statements in section 3.3.

Statement	Proposed level
The choice of cTACE, DEB-TACE, or a combined approach should depend on tumor vascularity, tumor size, individual patient characteristics, and facility capabilities	Level B
cTACE may be preferred in selected tumors within the “up-to-7” criterion	Level D
DEB-TACE may be preferred in selected tumors outside the “up-to-7” criterion and/or in patients with impaired liver function	Level C
A combination of cTACE and DEB-TACE may be considered on a case-by-case basis	Level D
Commonly used chemotherapeutic agents include doxorubicin, epirubicin, famorubicin, cisplatin, 5-fluorouracil, and absolute alcohol, with dosages individualized according to patient characteristics	Level D
Occlusion of tumor-feeding arteries with temporary embolic materials may be performed following cTACE	Level B
Lipiodol may be used in cTACE, generally with a maximum dose of 15 mL per session, mixed with chemotherapeutic agents at an appropriate ratio and prepared according to proper technique	Level B
Radioembolization using microspheres may be considered across different HCC stages, including selected advanced cases with portal vein or hepatic vein invasion	Level B
Bead size should be selected according to tumor size, degree of selectivity, and microcatheter size; beads ranging from 100–300 μm are commonly used	Level C
Chemotherapeutic agents compatible with drug-eluting beads should be selected appropriately; doxorubicin is commonly used	Level B
Microcatheter size should be selected according to the intended degree of selectivity and bead size	Level D
Selective access to subsegmental hepatic artery branches should be prioritized during DEB-TACE when feasible	Level B
DEB-TACE may be used for extrahepatic tumor-feeding arteries if superselective access can be achieved	Level D
DEB-TACE may be feasible in cases with hepatic arteriovenous shunts after adequate shunt control	Level D
Drug-eluting beads should be prepared before the procedure according to the manufacturer's instructions	Level D
Beads should be mixed with contrast medium and injected under DSA guidance to reduce the risk of reflux	Level D
Bead injection should be stopped when near-stasis is achieved, followed by reassessment after a short interval, with additional injection considered when appropriate	Level D
cTACE may be combined with DEB-TACE in selected situations	Level D
If vessel rupture occurs during bead injection, the procedure should be stopped and managed according to the severity and progression of hematoma	Level C

**Table 4:** Proposed level of support for statements in section 3.4.

Statement	Proposed level
Prophylactic antibiotics before TACE should be considered in high-risk patients	Level B
Intra-arterial selective lidocaine during TACE may reduce the incidence and severity of post-embolization symptoms	Level B
Antiemetic and anti-inflammatory prophylaxis may improve peri- and post-procedural symptoms and reduce the severity of post-embolization syndrome	Level B
Patients should be monitored closely to allow early detection of local and systemic complications after TACE	Level D
Post-embolization syndrome should be managed according to symptom severity with appropriate supportive care	Level D
Tumor response should be assessed 4–8 weeks after TACE using multiphasic contrast-enhanced CT and/or MRI	Level B
mRECIST or LI-RADS treatment response criteria, together with changes in tumor markers, should guide response assessment	Level B
TACE failure or refractoriness may be defined by the stated radiologic, biologic, and disease-progression criteria after at least two TACE sessions	Level B
TACE should be discontinued in cases of failure, refractoriness, or unsuitability, as determined by a multidisciplinary tumor board	Level B

**Table 5:** Proposed level of support for statements in section 3.5.

Statement	Proposed level
Pre-surgical TACE may be considered for intermediate-stage HCC localized to a lobe or segment in selected patients with adequate liver function and sufficient future liver remnant, as decided by a multidisciplinary tumor board	Level C
Post-surgical TACE may be considered for intermediate-stage recurrent HCC that is unsuitable for radiofrequency ablation or repeat surgery	Level C
Pre-surgical TACE may enhance treatment efficacy in selected surgical candidates	Level C
Portal vein embolization and/or hepatic vein embolization after TACE may induce hypertrophy of the future liver remnant and expand surgical eligibility in selected patients	Level B
Pre-ablation TACE may be considered for intermediate-stage HCC measuring >3 cm with ≤3 nodules, as decided by a multidisciplinary tumor board	Level B
Post-ablation TACE may be considered for recurrent intermediate-stage HCC that is unsuitable for further ablation or surgery	Level C
Pre-ablation TACE may reduce tumor size and improve lesion localization, thereby facilitating ablation and potentially reducing recurrence	Level B
TACE may enhance tumor conspicuity on ultrasound or CT, thereby facilitating local ablation procedures	Level C
TACE plus systemic therapy may be considered for intermediate-stage HCC at high risk of refractoriness and for selected advanced HCC with liver-confined disease, as decided by a multidisciplinary tumor board	Level B
Combination therapy may reduce post-TACE progression and extend the interval between TACE sessions, thereby helping preserve liver function	Level B
Systemic therapy should be initiated after TACE failure or when TACE is no longer feasible	Level B
TACE may be considered after a favorable response to HAIC in an effort to achieve complete response in selected patients	Level D
TACE may be combined with HAIC in selected situations, particularly when extrahepatic feeding arteries emerge and may reduce the effectiveness of HAIC alone	Level D
Non-port HAIC together with TACE may be considered in selected patients with high intrahepatic tumor burden and/or portal vein tumor thrombus	Level C

## 4 Discussion

This Vietnamese expert consensus was developed to address the need for a more standardized and context-adapted approach to transarterial chemoembolization (TACE) for hepatocellular carcinoma (HCC) in Vietnam. Although international guidelines provide an important evidence-based foundation, their application in daily practice may vary across institutions because of differences in infrastructure, access to advanced imaging and embolization materials, multidisciplinary coordination, and operator experience. In Vietnam, these differences are particularly relevant between tertiary referral centers and provincial hospitals, where technical resources and interventional radiology expertise may not be uniform. Therefore, a national expert consensus may help bridge the gap between international evidence and real-world clinical practice.

A major strength of this document is its multidisciplinary and practice-oriented nature. The recommendations were developed through structured literature synthesis, expert discussion, and consensus voting involving specialists from interventional radiology, gastroenterology/hepatology, hepatobiliary-pancreatic surgery, and medical oncology. This approach allowed the recommendations to reflect not only published evidence but also practical considerations encountered in routine HCC care, including patient selection, imaging work-up, technical execution, peri-procedural management, and integration of TACE with other treatment modalities. As such, this consensus is intended to serve as a practical reference for interventional radiologists and multidisciplinary teams involved in HCC treatment in Vietnam.

This document also has several limitations. First, it represents an expert consensus rather than a formal national guideline, and the literature review was structured to support discussion and statement development rather than conducted as a full systematic review. Second, some recommendations were necessarily influenced by expert experience and local feasibility, particularly in areas where high-level evidence remains limited or where practice varies across centers. Third, as HCC management continues to evolve rapidly, especially in relation to combination therapies, response-adapted strategies, and emerging technologies, some recommendations may require refinement as new evidence becomes available.

For these reasons, this consensus should be regarded as a dynamic practice document. Future updates will be needed to reflect new clinical evidence, technological advances, and the evolving treatment landscape of HCC. Periodic revision will be particularly important for areas such as TACE patient selection, treatment sequencing, multimodality therapy, and imaging-guided technical optimization.

## 5 Conclusion

This Vietnamese expert consensus provides a practical and context-adapted framework for the use of TACE in HCC. It is intended to support greater standardization of TACE practice in Vietnam and to serve as a practical reference for interventional radiologists and multidisciplinary teams. Given the rapidly evolving evidence base and treatment landscape of HCC, these recommendations should be considered a dynamic consensus document and updated regularly as new evidence and clinical experience emerge.
